# Molecular and immunological analyses of confirmed *Plasmodium vivax* relapse episodes

**DOI:** 10.1186/s12936-017-1877-x

**Published:** 2017-05-30

**Authors:** Sarunya Maneerattanasak, Panita Gosi, Srivicha Krudsood, Pattamawan Chimma, Jarinee Tongshoob, Yuvadee Mahakunkijcharoen, Chonlaphat Sukasem, Mallika Imwong, Georges Snounou, Srisin Khusmith

**Affiliations:** 1 Department of Microbiology and Immunology, Faculty of Tropical Medicine, Mahidol University, 420/6 Rajvithi Road, Bangkok, 10400 Thailand; 2 Department of Immunology and Medicine, Armed Forces Research Institute of Medical Science-United States Army Military Component, Bangkok, Thailand; 3 Clinical Malaria Research Unit, Faculty of Tropical Medicine, Mahidol University, Bangkok, Thailand; 4 Office for Research and Development, Faculty of Medicine Siriraj Hospital, Mahidol University, Bangkok, 10700 Thailand; 5 Division of Pharmacogenomics and Personalized Medicine, Department of Pathology, Faculty of Medicine Ramathibodi Hospital, Mahidol University, Bangkok, 10400 Thailand; 6 Laboratory for Pharmacogenomics, Somdech Phra Debaratana Medical Center (SDMC), Ramathibodi Hospital, Bangkok, Thailand; 7 Department of Molecular Tropical Medicine and Genetics, Faculty of Tropical Medicine, Mahidol University, Bangkok, 10400 Thailand; 8 UPMC Univ Paris 06, Inserm (Institut National de la Santé et de la Recherche Medicale), Centre d’Immunologie et des Maladies Infectieuses (Cimi-Paris), UMR 1135, ERL CNRS 8255 (Centre National de la Recherche Scientifique), Sorbonne Universités, 91 Boulevard de l’Hôpital, 75013 Paris, France; 9 Center for Emerging and Neglected Infectious Diseases, Mahidol University, Bangkok, Thailand

**Keywords:** Antibodies, Cytokines, Genotyping, Relapse, *Plasmodium vivax*, Thailand

## Abstract

**Background:**

Relapse infections resulting from the activation hypnozoites produced by *Plasmodium vivax* and *Plasmodium ovale* represent an important obstacle to the successful control of these species. A single licensed drug, primaquine is available to eliminate these liver dormant forms. To date, investigations of vivax relapse infections have been few in number.

**Results:**

Genotyping, based on polymorphic regions of two genes (*Pvmsp1*F3 and *Pvcsp*) and four microsatellite markers (MS3.27, MS3.502, MS6 and MS8), of 12 paired admission and relapse samples from *P. vivax*-infected patients were treated with primaquine, revealed that in eight of the parasite populations in the admission and relapse samples were homologous, and heterologous in the remaining four patients. The patients’ CYP2D6 genotypes did not suggest that any were poor metabolisers of primaquine. Parasitaemia tended to be higher in the heterologous as compared to the homologous relapse episodes as was the IgG3 response. For the twelve pro- and anti-inflammatory cytokine levels measured for all samples, only those of IL-6 and IL-10 tended to be higher in patients with heterologous as compared to homologous relapses in both admission and relapse episodes.

**Conclusions:**

The data from this limited number of patients with confirmed relapse episodes mirror previous observations of a significant proportion of heterologous parasites in relapses of *P. vivax* infections in Thailand. Failure of the primaquine treatment that the patients received is unlikely to be due to poor drug metabolism, and could indicate the presence of *P. vivax* populations in Thailand with poor susceptibility to 8-aminoquinolines.

**Electronic supplementary material:**

The online version of this article (doi:10.1186/s12936-017-1877-x) contains supplementary material, which is available to authorized users.

## Background

The origin of renewed parasitaemia following a primary infection by *Plasmodium vivax* or *Plasmodium ovale* treated with a schizontocidal drug is either a recrudescence due to erythrocytic parasites that have survived treatment, a re-infection, or a relapse consequent to merozoites released from a dormant liver stage, the hypnozoite, that has activated to resume normal development. Hypnozoites, which are not detectable, significantly extend the duration of the infection and the parasite’s potential to transmit, as a single infected mosquito bite can lead to multiple relapse episodes over many years. Relapse patterns vary between the parasite from temperate and tropical origin, and the risk for multiple relapse is highest in the tropics (50–80%) and lowest in temperate regions (5–10%) [1]. In Southeast Asia, vivax infections are predicted to have a high relapse incidence rate (836 relapses per 100,000 person days) and a relatively rapid mean time from primary episode to first relapse (41 days) [2]. The mechanisms behind the activation of hypnozoites and the determination of their periodicity remain unclear. Hypnozoites can only be eliminated by 8-aminoquinolines of primaquine which is safely administered only to persons without glucose-6-phosphate dehydrogenase deficiency. Thus, relapses are a major concern for efforts to control and eliminate vivax malaria [3].

It was assumed that the parasite populations emerging during relapses would be identical to those present during the primary episode, a notion that received support from early molecular studies [4, 5]. However, later investigations based more detailed genotyping analyses on relapses in diverse geographic regions revealed that the parasites in primary and relapse episodes are often genetically distinct, a phenomenon termed heterologous hypnozoite activation [6–8]. Thus, interpretation of drug efficacy studies against *P. vivax* in endemic countries cannot be corrected by genotyping analyses, as these will not allow distinguishing between a re-infection, a recrudescence, or a heterologous relapse.

The relapse pattern comprising the latency period and the number of relapse episodes is probably influenced by the level of immunity that the patients may have acquired during the primary attack [1, 9]. A degree of immunity is generally acquired following the primary infection, and experimental vivax infections clearly show that protection is strain-specific [9–11]. However, although the number of investigations on the immune responses in *P. vivax* is increasing, very few studies have addressed this in the context of relapse episodes [5, 12–16].

In Thailand patients diagnosed with *P. vivax* receive a standard treatment of chloroquine and primaquine, though approximately 7% relapse within 6 months [17]. Patients who present with *P. vivax* in Bangkok following a trip to endemic areas cannot be re-infected while they remain there, as malaria transmission does not occur in this city. This afforded the opportunity to recruit a cohort of vivax patients and to identify those with true relapse episodes during extended follow-up. In this manner a set 12 paired admission/relapse blood samples were obtained, and these were used to compare the genotypes of the parasites and a set of immune response markers.

## Methods

### Samples

The stored packed red blood cells and plasma samples were obtained from Clinical Malaria Research Unit, Faculty of Tropical Medicine at Mahidol University (Bangkok, Thailand). The samples were collected between February 2012 and May 2013 prior to treatment by the Clinical Malaria Research Unit (Faculty of Tropical Medicine) from 12 patients (P1 to P12) on initial admission with *P. vivax* and when presenting with renewed clinical symptoms during follow-up. These infections were acquired while visiting malaria endemic areas along the Thai-Myanmar border in the west (P1, P2, P3, P4, P5, P6, P7, P9), the Thai–Cambodian border in the east (P10, P11), and the Thai-Lao border in the north (P8, P12). All patients were treated the standard chloroquine/primaquine treatment: once-daily oral chloroquine on day 1 (600 mg), day 2 (600 mg), and day 3 (300 mg), and oral primaquine 15 mg for 14 days from day 2.

The mean age (mean ± SD) of the patients was 29.8 ± 13.3 years. The admission episode was confirmed to be the first following the latest travel to malarial areas through an interview of the patient and an examination of their clinical record. The second episode was confidently classed as a true relapse because: (a) reinfection could be excluded since the relapse episode occurred when the patients were residents in or around Bangkok, a non-malaria area, without any history of travelling to any malaria endemic areas after the initial *P. vivax* episode; (b) the median time interval to relapse in these patients was 81 days, making it highly unlikely that this was a recrudescence, which usually occurs within 28 days after treatment [18].

Thirty anonymous frozen plasma samples obtained from healthy individuals resident in non-endemic areas were used as controls. These donors had no history of malaria and denied travelling to any endemic area during the 2 years preceding blood collection.

This study was approved by the Ethics Committee of the Faculty of Tropical Medicine, Mahidol University (Approval Number: MUTM 2013-034-01).

### DNA template preparation

*Plasmodium vivax* genomic DNA was extracted from 200 µL of frozen-packed red cells using the commercially available DNA Blood kit (MACHEREY–NAGEL, Germany). The final volume of the DNA template was 100 µL, thus each 1 µL corresponds to the DNA present in 2 µL of whole blood. Confirmation of microscopic diagnosis examination was achieved by a multiplex real-time PCR assay using both genus- and species-specific primers [19].

### Genotyping of *Pvmsp1*F3 and *Pvcsp* genes

Polymorphic fragments of two genes: F3 fragment of the *P. vivax* merozoite surface protein 1 (*Pvmsp1*F3) and the central repeat domain of the *P. vivax* circumsporozoite protein (*Pvcsp*) were amplified by nested-PCR assay as previously described [20]. The PCR products were purified using the QIAquick Gel Extraction Kit (QIAGEN, Hilden, Germany) and sequenced by AIT Biotech, Singapore. The sequences were aligned and analysed using Sequencher™ software version 5.3 (Gene Codes Corporation, Ann Arbor, USA).

### Microsatellite analysis

Four microsatellite (MS) markers (MS3.502, MS3.27, MS6, and MS8) were amplified using semi-nested PCR amplification as previously described [21, 22]. The length variation of labelled PCR products was measured in comparison to internal size standards (GeneScan 500 LIZ; Applied Biosystems) on an ABI Genetic Analyzer (Macogen Inc., South Korea). Allele lengths and peak heights were analysed by using PeakScanner software (Applied Biosystems). Multiple alleles were called when there were multiple peaks per locus and where the height of these minor peaks reached one-third or more (>33%) of the predominant allele peak height.

### Detection of anti-*Plasmodium vivax* IgG and IgG subclasses antibody

The anti-*P. vivax* IgG, IgG1, IgG2, IgG3, and IgG4 antibodies levels in the plasma samples were determined by the enzyme-linked immunosorbent assay as previously described [23] with some modification using crude *P. vivax* blood-stage antigens. The optical density (OD) was measured at a wavelength of 450 nm by an ELISA plate Reader (Tecan Sunrise™, Austria) with Magellan™ data analysis software version 7.1 (Tecan, Austria). Plasma from normal healthy individuals was used as controls.

### Plasma cytokine quantification

The plasma pro- and anti- inflammatory cytokines were quantified by human cytometric bead array (CBA) kits (BD Biosciences Pharmingen, San Jose, CA, USA) for IL-1β (interleukin-1 beta), IL-6, IL-8, IL-10, IL-12p70 and TNF (tumour necrosis factor), and by CBA Flex Set system (BD Biosciences Pharmingen, San Jose, CA, USA) for IL-2, IL-4, IL-5, IL-13, IL-17A and IFN-γ (interferon gamma). Standard curves for each cytokine were generated by using the reference cytokine concentrations supplied with the kits. All standards and plasma samples were acquired on Accuri™ C6 Plus personal flow cytometer (BD Biosciences). Data analysis was performed using the FCAP Array software version 3.0.1 (BD/Softflow, Pécs, Hungary) and the values were expressed in pg/mL.

### *CYP2D6* genotyping and phenotyping

The single-nucleotide polymorphisms (SNPs) in the human gene encoding the cytochrome P450 2D6 (*CYP2D6*) were determined using the Luminex xTAG CYP2D6 Kit v3 (Luminex Corporation). The assay identifies the following alleles: normal function *1, *2, *35; decreased function *9, *10, *17, *29, *41; no enzymatic function *3, *4, *5, *6, *7, *8, *11, *15, and duplications. The CYP2D6 metaboliser phenotypes were assigned according to the rule-base system of Luminex xTAG CYP2D6 Kit v3 [24]. The patients were classified as (1) poor metabolisers (PM) if they harboured two null (non-functional) alleles; (2) intermediate metabolisers (IM) if they harboured two decreased function alleles, or a null allele with a decreased function allele; (3) extensive metabolisers (EM) if they harboured two functional alleles, or a functional allele with either a decreased function allele or a null allele; (4) ultra-rapid metabolizer (UM) if they harboured duplication of functional alleles.

### Statistical analyses

Statistical analyses were performed using SPSS 15.0 software (SPSS Inc, Chicago, IL, USA) and Prism v.5 (GraphPad Software Inc, San Diego, CA, USA). Dimension variables were compared using Wilcoxon signed rank test for dependent data and Mann–Whitney *U* test for independent data. The *P* value of <0.05 was considered as statistically significant.

## Results

### Homologous *versus* heterologous relapses

Genotyping based on the *Pvmsp1*F3 and *Pvcsp* was successfully achieved for the parasites in all twelve paired admission and relapse samples (Table 1). Six distinct allelic variants (designated as “*a*” to “*f*”) were observed for *Pvmsp1*F3, and 13 distinct allelic variants were observed for *Pvcsp*: nine of the VK210 type designated as “*a”* to *“i*”, and four genotypes of the VK247 type designated as “*j”* to *“m*”. The sequences of the allelic variants are presented in Additional file 1. The parasites in the admission and relapse samples were also successfully genotyped using four microsatellite (MS) markers: MS3.27, MS3.502, MS6 and MS8 (Table 1). The number of distinct allelic variants for these microsatellite markers ranged from 6 to 17. Mixed genotype infections were observed in one or both samples from seven of the patients, with a maximum of two distinct haplotypes detected in a given sample (Table 1).

**Table 1:** Genotypes of the *P. vivax* populations in admission and relapse episodes

Patient/age (days)^a^	Date of sample collection (D/M/Y)	*Msp1*F3	*Csp*	Microsatellite			
				MS3.27	MS3.502	MS 6	MS 8
P1/47	AS (12/3/2012)	*a*	VK247 *j*	110.7	150.7	252.3	243.7
(106)	RS (25/6/2012)	*a*	VK247 *j*	110.7	150.6	252.3	243.7
P2/56	AS (12/3/2012)	*a*	VK247 *j*	110.7	150.7	252.2	243.7
(120)	RS (09/7/2012)	*a*	VK247 *j*	110.7	150.6	252.3	243.7
P3/50	AS (12/3/2012)	*a*	VK247 *j*	110.7	150.7	252.3	243.7
(106)	RS (25/6/2012)	*a*	VK247 *j*	110.6	150.6	252.3	243.7
P4/18	AS (23/4/2012)	*a*	VK247 *j*	110.7	150.6	252.3	243.7
(44)	RS (05/6/2012)	*a*	VK247 *j*	110.8	150.7	252.3	243.8
P5/20	AS (22/5/2012)	***a***	**VK247** ***k***	**95.5**	**150.6**	252.3	**311.0** **+** **314.0**
(38)	RS (28/6/2012)	***b***	**VK210** ***a***	**125.9**	**190.9**	252.2	**304.8** **+** **307.8**
P6/22	AS (02/7/2012)	*c*	VK210 b	103.1	158.8	240.4	311.1 + 314.1
(120)	RS (29/10/2012)	*c*	VK210 b	103.2	158.8	240.3	311.1 + 314.2
P7/22	AS (16/7/2012)	*a*	VK210 *c*	129.8	166.8	252.1	223.8
(92)	RS (15/10/2012)	*a*	VK210 *c*	129.7	166.9	252.2	223.9
P8/31	AS (07/2/2013)	*a*	**VK210** ***d*** + VK247 *l*	**118.2**	**158.8**	**246.1**	**249.5**
(67)	RS (13/4/2013)	*a*	VK247 *l*	**185.9**	**150.7**	**240.3**	**284.3** **+** **287.3**
P9/22	AS (07/8/2013)	*d*	VK210 *e*	146.3	141.7	240.4	272.7 + 275.5
(68)	RS (13/10/2013)	*d*	VK210 *e*	146.4	141.7	240.3	272.7 + 275.6
P10/22	AS (18/5/2013)	*a* + ***e***	**VK210** ***f***	133.8	133.1	243.2 + **246.3**	**287.1** **+** **289.9**
(153)	AS (17/10/2013)	*a*	**VK210** ***g***	133.7	133.1	243.16	**218.2**
P11/21	AS (11/9/2013)	*a*	**VK210** ***h*** + VK247 *m*	118.2	141.7	**246.2**	229.5
(40)	RS (20/10/2013)	*a*	**VK210 ** ***i*** + VK247 *m*	**110.6** + 118.2	141.7	**240.3**	229.5 + **246.7**
P12/27	AS (23/10/2013)	*f*	VK210 *b*	150.6	190.9	261.2	278.5 + 281.4
(69)	RS (30/12/2013)	*f*	VK210 *b*	150.6	191.0	261.2	278.4 + 281.4

Relapse episodes were classified as heterologous when a new allelic variant was observed in this episode for at least one of the markers

*AS* admission sample, *RS* relapse sample. Allelic variants that differ between the paired samples from each patient are presented in bold

^a^The number of days between the initial admission and the relapse episode is presented in parenthesis below each patient number

The genotyping data allowed classifying the relapse episodes as homologous or as heterologous, i.e. with genetically similar or distinct parasite populations in the paired samples, respectively. For eight patients (P1, P2, P3, P4, P6, P7, P9 and P12) the relapses were homologous, while for the other four they were clearly heterologous (Table 1).

**Fig. 1:**
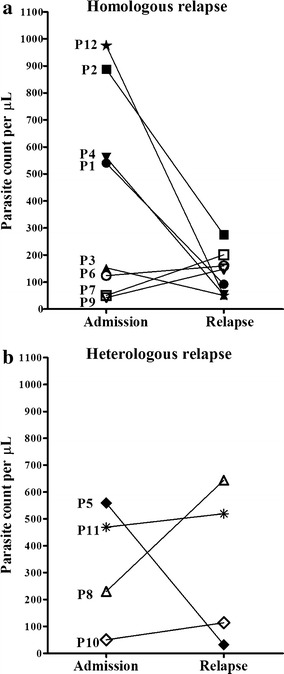
Parasitaemia in admission and relapse episodes of patients with homologous and heterologous relapse. Each *line* represents data from one patient. Parasite numbers per microliter of blood were determined by counting the number of parasites per 200 white blood cells (WBCs) in thick films and assuming an average of 8000 WBC/µL of blood to calculate the parasitaemia (P/µL of blood)

Median parasitaemia at first presentation was quite similar for the patients who then had a heterologous relapse (median and interquartile range: 349.3, 94.9–536.5 parasites/µL) as compared to those who went on to have a homologous relapse (346.5, 68.5–806.3 parasites/µL). On the other hand, the median parasitaemia at presentation of the relapse episode was higher in those with heterologous relapse (316.3, 52.1–612.3 parasites/µL) as compared to those with a homologous relapse (119.8, 50.4–190.8 parasites/µL) (Fig. 1).

### *CYP2D6* genotyping of the patients

The twelve patients recruited in this study had received primaquine in addition to chloroquine as treatment for the initial admission episode. Their relapse episodes could therefore be considered as hypnozoicidal treatment failures. Given that adherence to treatment was observed, failure could be due to reduced susceptibility to 8-aminoquinolines or an inability of the patients to metabolise primaquine to its active form. Recent studies showed that cytochrome P450 2D6 (CYP2D6) is implicated in the metabolism of primaquine [25]. CYP2D6 is a genetically and phenotypically highly polymorphic drug-metabolizing enzyme involved in the biotransformation of numerous drugs [26]. Indeed, two cases where primaquine failure was associated to carriage of a particular CYP2D6 haplotype have been recorded [27, 28].

The CYP2D6 genotypes and the predicted metaboliser phenotypes were obtained for the patients recruited in this study (Table 2). Eight of the patients were classed as extensive metabolisers (EM) while the remaining four were predicted to be intermediate metabolisers (IM), and their proportion in those who had a homologous relapse (3 IM/5 EM) was similar to that in those who experienced a heterologous relapse (Table 2).

**Table 2:** *CYP2D6* genotypes and predicted phenotypes

Relapse	Patient	Drug treatment	Genotype	Predicted phenotype
Homologous	P1	C/P	*5/*10	IM
	P2	C/P	*10/*10	IM
	P3	C/P	*1/*41	EM
	P4	C/P	*2/*10	EM
	P6	C/P	*1/*1	EM
	P7	C/P	*10/*10	IM
	P9	C/P	*1/*10	EM
	P12	C/P	*1/*10	EM
Heterologous	P5	C/P	*2/*10	EM
	P8	C/P	*1/*10	EM
	P10	C/P	*10/*41	IM
	P11	C/P	*1/*2	EM

*C/P* chloroquine/primaquine, *IM* intermediate metabolisers, *EM* extensive metabolisers

### Analysis of immune responses

**Fig. 2:**
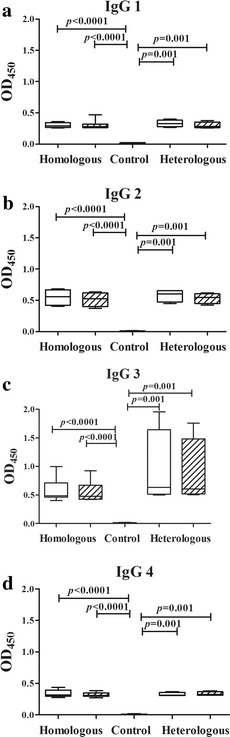
Levels of *Plasmodium vivax*-specific IgG subclasses in plasma collected at admission and relapse. The admission and relapse episodes are represented by *white* and *crosshatched bars*, respectively, while normal controls are indicated by a *black bar*. The *box plots* show the median (50th percentile) and 25th and 75th quartiles; *error bars* show the 10th and 90th percentiles. Mann–Whitney *U* test was used to establish a significant difference (*P* < 0.05) between groups pairwise. Wilcoxon signed rank test was used to assess the significant differences in the specific IgG subclass levels between the admission and relapse episodes

Anti-*P. vivax* antibody levels (IgG, IgG1, IgG2, IgG3 and IgG4) were measured in the plasma samples collected from the patients on for the initial and the relapse episodes (Fig. 2). As expected the median levels (median and interquartile range) of the specific IgG, IgG1, IgG2, IgG3 and IgG4 antibodies in both episodes were significantly higher than those in normal controls (*P* < 0.05) for all the patients. Significant differences in total IgG, IgG1, IgG2, IgG3 and IgG4 levels in paired admission and relapse episodes were not observed. This was also the case when considering samples from patients with homologous as compared to heterologous relapses, except for IgG3 where the median levels observed in the paired samples tended to be higher in patients who experienced a heterologous relapse (Fig. 2c).

At both admission and relapse episodes, the median IgG3 levels in patients with heterologous relapses (admission: 0.63, 0.51–1.64; relapse: 0.61, 0.52–1.48) were relatively higher than those with homologous relapse (admission: 0.49, 0.47–0.72; relapse: 0.48, 0.43–0.67) but these were not statistically significant (*P* > 0.05) (Fig. 2c).

**Fig. 3:**
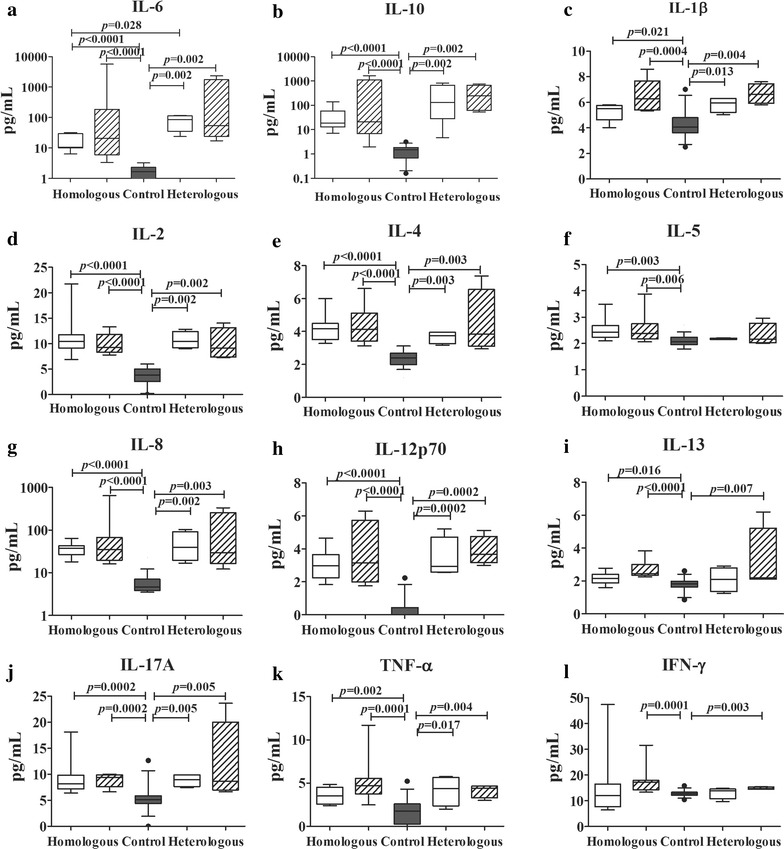
Levels of cytokines in plasma collected at admission and relapse. The admission and relapse episodes were indicated by *white* and *crosshatched bars*, respectively, while the normal control was indicated by *black bar*. The *box plots* show the median (50th percentile) and 25th and 75th quartiles; *error bars* show the 10th and 90th percentiles. Mann–Whitney *U* test was used to establish a significant difference (*P* < 0.05) between groups pairwise. Wilcoxon signed rank test was used to assess the significant differences in cytokine and chemokine levels between the admission and relapse episodes

The cytokine profiles were similarly measured in the plasma samples collected from the patients prior to treatment (Fig. 3). As expected, the median cytokine levels (median and interquartile range) in the admission and relapse episodes were higher levels than those in normal controls. No Differences in median levels were observed for IL-2, IL-4, IL-5, IL-8, IL-12p70, IL-13, IL-17A, TNF or IFN-γ in either patient groups (Fig. 3). IL-6 (Fig. 3a) and IL-10 (Fig. 3b) were higher in the paired samples collected from patients with heterologous relapses as compared with those who had a homologous relapse with a significant difference for IL-6 at admission [heterologous versus homologous: 86.2, 35.4–113.3 pg/mL versus 10.7, 10.1–29.6 pg/mL (*P* = 0.028)], while significant differences were not seen for IL-6 at relapse [54.7, 23.8–1762.9 pg/mL versus 21.2, 6.1–187.0 pg/mL (*P* = 0.125)], or for IL-10 for either samples [admission: 130.6, 28.4–666.3 pg/mL versus 18.9, 13.0–58.9 pg/mL (*P* = 0.283); relapse: 252.5, 62.4-670.2 pg/mL versus 20.9, 6.9–1118.2 pg/mL (*P* = 0.283)]. IL-1β levels tended to be higher in the relapse as compared to the admission sample for both homologous [relapse versus admission: 6.3, 5.4–7.7 pg/mL versus 5.5, 4.6–5.8 pg/mL (*P* = 0.078)] and heterologous relapses [6.6, 5.9–7.4 pg/mL versus 6.0, 5.2–6.3 pg/mL (*P* = 0.375)] (Fig. 3c).

## Discussion

The biology of hypnozoites remains very poorly investigated; indeed the few hypnozoites consequent to an infectious bite lie dormant in the liver, an organ of a mean weight of 1.5 kg in adults, where they are not amenable to experimental in vivo investigations. Thus, insights into these dormant stages are only practicably inferred indirectly through observations of the relapses they cause. The ability of hypnozoites to survive for months and years and for a subset to activate rather randomly during this time makes this task difficult. In areas where transmission occurs, it is difficult to distinguish a true relapse episode from a recrudescence or a primary episode from a new infection during the follow-up of recruited patients. However, if the patients are treated with an efficacious schizontocidal treatment and observed in areas where transmission does not occur, one can confidently consider any renewed parasitological episodes as true relapses. The first molecular analyses of paired admission/relapse samples were carried out on samples from 6 patients who acquired the infection in various endemic countries who were treated and sampled in Canada [4], and the second from 10 patients recruited in an endemic region of Brazil [5]. In nearly all these cases the parasites in the paired samples were similar, but there were two exceptions (one in each study) where the parasite genotypes differed. The next molecular investigations on relapses that were conducted on a large number of patients, who had acquired their infection in Indonesia [6], Thailand, Myanmar or India [7], clearly demonstrated that the activation of heterologous hypnozoites could actually be quite frequent. Similar subsequent studies from diverse endemic areas confirmed that this phenomenon is indeed common.

Variations in the proportion of heterologous relapses are likely to alter with the levels and the duration of exposure to transmission, as this will influence the number of hypnozoites that accumulate in the liver. Of the 36 patients infected in western Thailand between 1992 and 1998 [7], 28 (78%) had parasite genotypes on relapse that were not observed in the admission sample. In this current study, only four of the twelve (33%) patients recruited between 2012 and 2013 showed a heterologous relapse, a decrease that might reflect the consistent reduction in the transmission intensity of *P. vivax* in this area (<0.5 infectious bite per person per year). It should be noted that both patients infected at the eastern border and one of the two infected at the north-eastern border showed heterologous relapses, as compared to one of the eight who acquired the infection at the western border of Thailand. Indeed, along the border areas of Thailand, it has been reported that *P. vivax* genetic diversity and multiplicity of infection remain high despite low malaria transmission [7, 29, 30]. The proximity to the borders of Myanmar, Laos and Cambodia (all areas where malaria endemicity is higher than in Thailand) has probably contributed to increase diversity through the extensive human migration events to and from Thailand. As the genetic diversity in the background population was high, the probability of relapses likely reflects heterologous infections. A larger number of cases would need to be investigated to ascertain whether this represents a significant difference. Nonetheless, the high genetic diversity of the parasites observed in the limited number of samples analysed here, with unequivocal mixed genotype infections in three of the patients, underlines the resilience to standard control efforts that hypnozoites confer to *P. vivax*.

It is interesting to note that patients P1–P4 had parasites of the same genotype (in both admission and relapse samples). These four patients were admitted within a short period of each other (March/April 2012), making it plausible that were infected by the same strain. The short time to relapse in one of these patients (P4, 44 days) compared to the other three (P1–P3, 106–120 days) might reflect age differences in the level of acquired immunity [31] as P4 was 18 years old whereas P1–P3 were 47–46 years old. Nonetheless, large variations in the time to relapse have been observed in humans inoculated experimentally with *P. vivax* sporozoites [32, 33]. An earlier relapse might also be due to a febrile illness that was proposed as a potential activator of latent hypnozoites [1]. It should be noted that a clinically discrete or silent relapse would have been missed because the study protocol relied on presentation with clinical symptoms during the follow-up period in order to record a relapse episode.

The patients presented here failed to clear the hypnozoites they were carrying despite the primaquine treatment that they had received. This failure was not related to a reduced ability to metabolise 8-aminoquinolines by cytochrome P450 as a result of CYP2D6 polymorphisms, reflecting previous observations showing similar clearance rates for intermediate and extensive metabolisers [34]. This suggests that some *P. vivax* lines circulating in Thailand might not be fully susceptible to the standard dose of primaquine (15 mg for 14 days from day 2), which should prompt reconsideration of the suitability of this dosage; all the more urgently should emergent chloroquine-resistant parasites [35] spread further. At present, there is no confirmed genetic marker for primaquine resistance/tolerance, though potential mutations in some parasite genes have been identified in the relapse populations of a single patient [36]. Whether any of these mutations are indeed implicated awaits comparative analysis of *P. vivax* isolates that have led or not to relapse infections following primaquine administration.

The availability of plasma samples from the patients afforded the possibility to conduct a survey of immune responses that could then be compared between admission and relapse episodes and also between homologous and heterologous episodes. Although the overall number of patients is low, particularly in the two relapse type groups, such studies have been previously rarely carried out and the comparisons could usefully serve to indicate factors that might be worth considering in future studies with larger patient groups. The levels of the twelve pro- and anti-inflammatory cytokines were as expected elevated in all groups as compared to controls, but differences between the groups were only noted for three of these: higher levels of IL-1β in the relapse as compared to the admission episode in patients with either relapse type, and elevated IL-6 and IL-10 in the paired samples from patients with heterologous as compared to homologous relapses. The significance of these observations is as yet unclear. Investigations on the cytokines profiles in *P. vivax* infections have to our knowledge been confined to admission and convalescent samples (for e.g. [37–40]) with a single study in which recurrences within 6 months, some of which might have been relapses, were included [12]. Given the pleiotropic effects of cytokines, and the diversity of epidemiological settings where the studies were based, conclusions from these studies remain speculative. Overproduction of IL-lβ is known to have deleterious effects on the liver [41]. Elevated levels of this cytokine are associated with the development of hepatic dysfunction in *P. vivax* malaria patients [40]. In this study, higher IL-1β levels in the relapse than admission episode might affect the development of the hypnozoite reservoir in the liver. Recently, it has been proposed that hypnozoites of different genotypes that have accumulated from previous inoculations could be triggered to activate by a febrile illness, leading to heterologous relapses [1]. Elevated IL-6 levels are associated with high fever in *P. vivax* malaria patients [39]. The higher IL-6 levels in heterologous as compared to homologous relapses in the current study might indicate the importance of this pyrogenic cytokine in the activation of heterologous hypnozoite. IL-10 has an important regulatory role in controlling the intensity of host immune response against the circulating parasites. Elevated IL-10 levels were shown to be correlated with hyperparasitaemia in *P. vivax* infected patients [42]. The high production of IL-10 noted for the patients with heterologous relapses suggests that it might be a regulator of immune responses harmful to the host.

Levels of IgG specific to *P. vivax* were elevated in all groups as compared to controls, which is consistent with previous observations on antibody levels [13, 16], and on the persistence of B cell memory responses to *P. vivax* blood stage antigens [43]. Previous studies have suggested that B cell memory responses to blood stage antigens could be stably maintained over time in the absence of reinfection even in areas of low malaria transmission [43]. The present investigation provides evidence that IgG antibody response to *P. vivax* was not greater in relapse compared with admission episodes even in patients with homologous relapses. This observation might be explained in three possibilities. Firstly, homologous relapses might not be associated with an enhanced immune memory-driven antibody response. It is possible that any partially effective immune response to a previous *P. vivax* strain might have been maximized in terms of driving elevations of antibody response [12]. Secondly, lower parasitaemia in homologous compared with heterologous relapses might be associated with a lower antibody response. Finally, some genotypes present in relapse episode may have been missed or only partially characterized due to insufficient numbers of parasites carrying those genotypes. Probably, in these patients with homologous relapses, the parasites causing relapses might not be identical to those of initial *P. vivax* infections. *P. vivax*-specific IgG2 and IgG3 levels were somewhat higher than those of IgG1 and IgG4 in all groups. It is not clear whether the higher median levels of IgG3 observed in both paired samples in heterologous relapses, as compared to those from homologous relapses, are of functional significance, especially as this does not correlate with the somewhat lower parasitaemia observed in homologous as compared to heterologous relapses. However, these observations might be explained by the possibility that the presence of genetically and antigenically distinct strains of the parasites during the course of infection might derive the host to mount diverse immune response against these antigenic variants. Higher parasite density may induce higher antibody responses in patients with malaria infection. In fact, IgG3 has the high binding affinity for the Fcγ receptor on the surface of monocytes, which strongly induces phagocytosis and complement fixing [44]. Recent studies determined that high IgG1 and IgG3 levels were associated with high parasite density [45].

## Conclusions

Insights into the biology of hypnozoites, the relapses they cause, and the immunological consequences of these persistent infections are needed to tailor control measures that would effectively reduce and then eliminate vivax malaria. The sampling strategy employed here ensures that true relapse cases are investigated, though only yielding relatively a small number of patients, particularly in areas with declining vivax prevalence. The molecular investigations on the nature of the relapses provides, nonetheless, a means to monitor the efficacy of the recommended first line treatments so as to provide early warning of failing drug dosages. Immunological investigations conducted on carefully selected patients in different endemic setting will be needed to address the specifics of acquired immunity to the *P. vivax* infections.

## Additional file

**Additional file 1.** Sequence alignment of *Pvmsp1*F3 and *Pvcsp* genes.
