# Supplementary material for: Molecular and immunological analyses of confirmed Plasmodium vivax relapse episodes
Source: Malar J. 2017 May 30;16:228. doi: 10.1186/s12936-017-1877-x (PMC5450361; doi:10.1186/s12936-017-1877-x)
Supplement: Supplementary file 1 — Additional file 1. Sequence alignment of Pvmsp1F3 and Pvcsp genes. [file 12936_2017_1877_MOESM1_ESM.docx]

**Additional file 1: Sequence alignment of *Pvmsp1*F3 and *Pvcsp* genes**


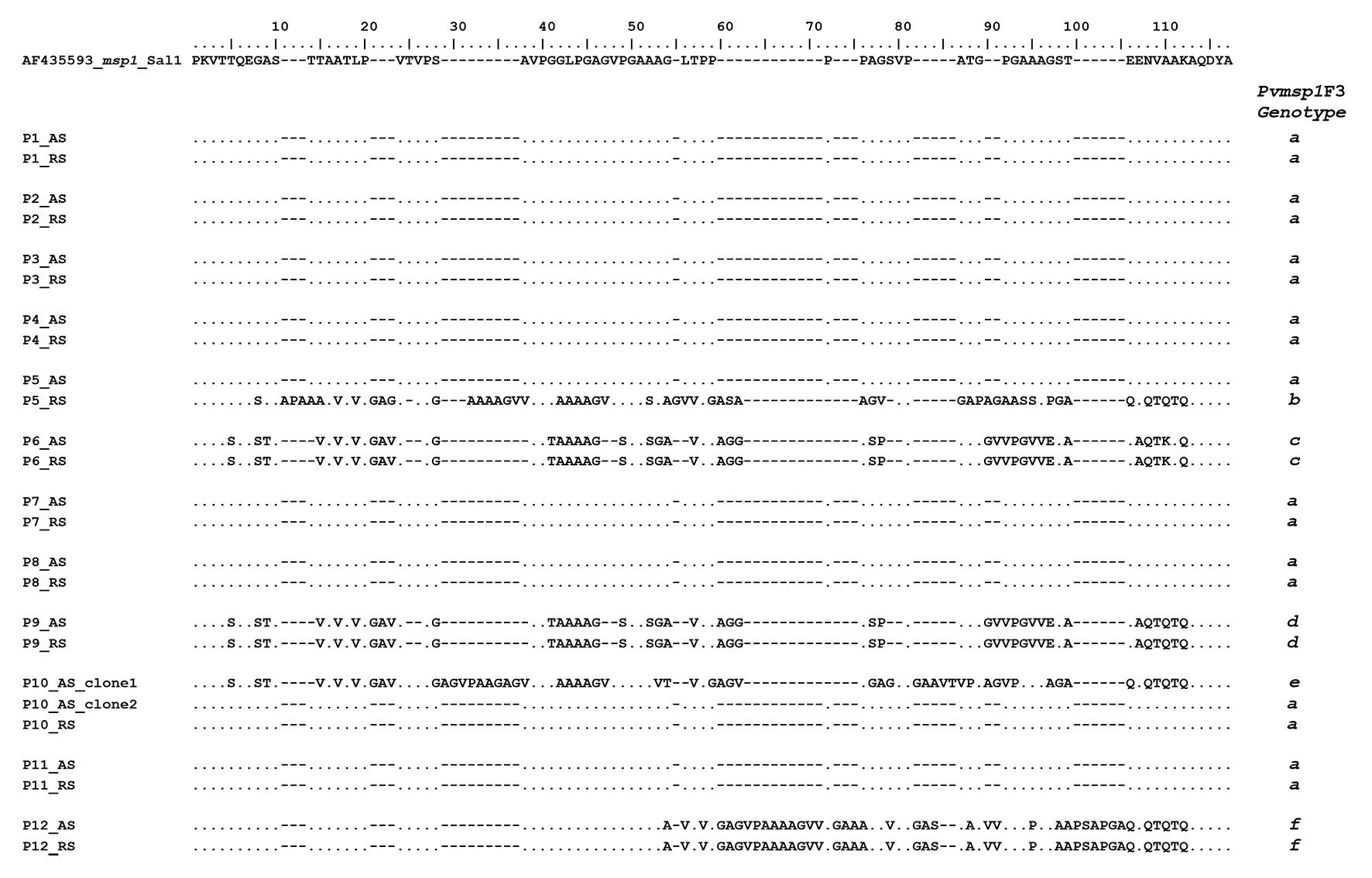


**Figure S1** Alignment of the predicted amino acid sequences of the *Pvmsp1*F3 fragments observed in the 12 paired admission and relapse samples. The alignment is based on the *Pvmsp1* sequence of the Salvador I strain (accession number AF435593). Dots and dashes indicate identical residues and deletion, respectively. P, patient; AS, admission sample; RS, relapse sample.

**
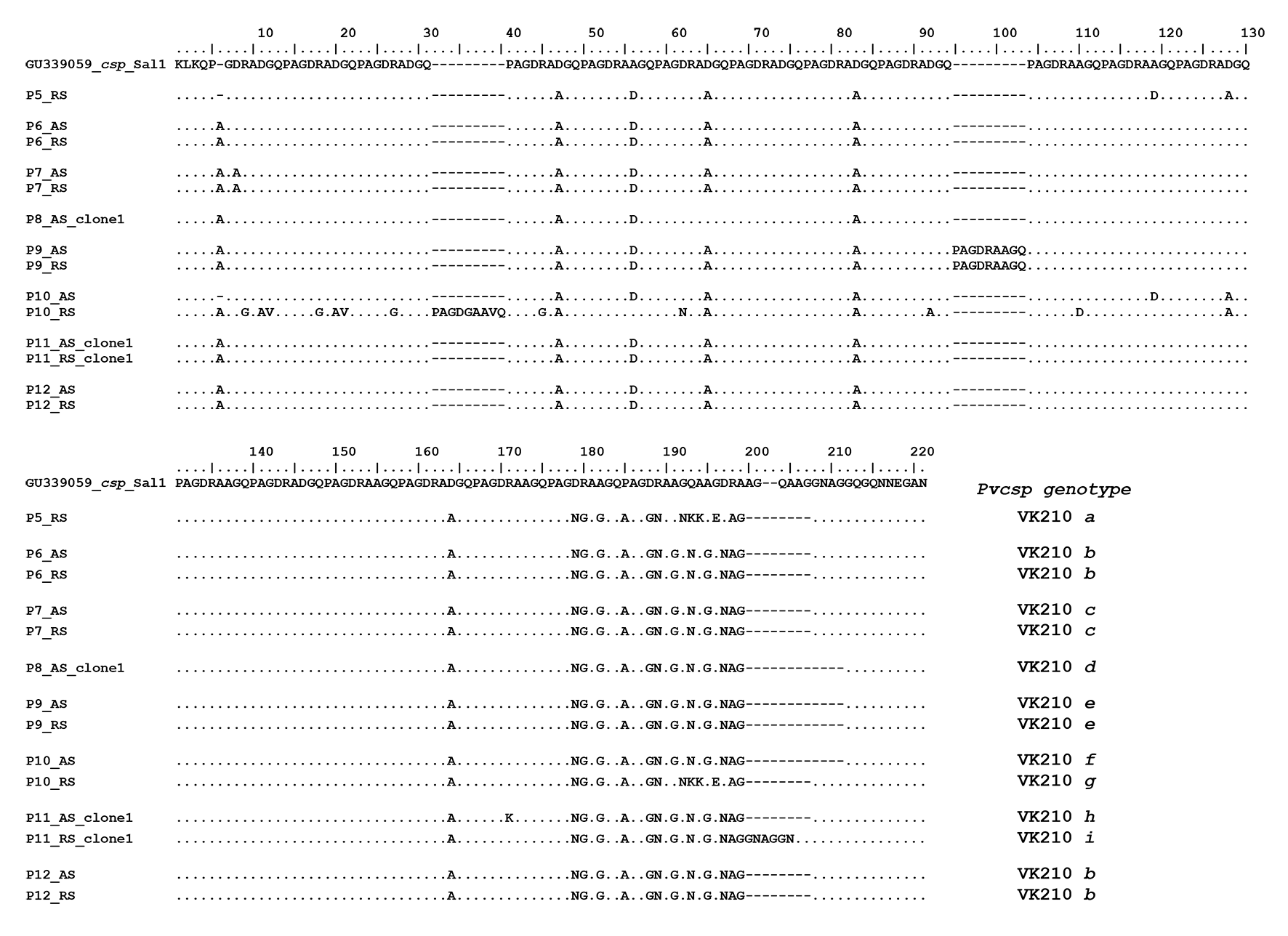
**

**Figure S2** Alignment of the predicted amino acid sequences of *Pvcsp* fragments bearing the VK210 repeat type observed in the 12 paired admission and relapse samples. The alignment is based on the *Pvcsp* sequence of the Salvador I strain (accession number GU339059). Dots and dashes indicate identical residues and deletion, respectively. P, patient; AS, admission sample; RS, relapse sample.


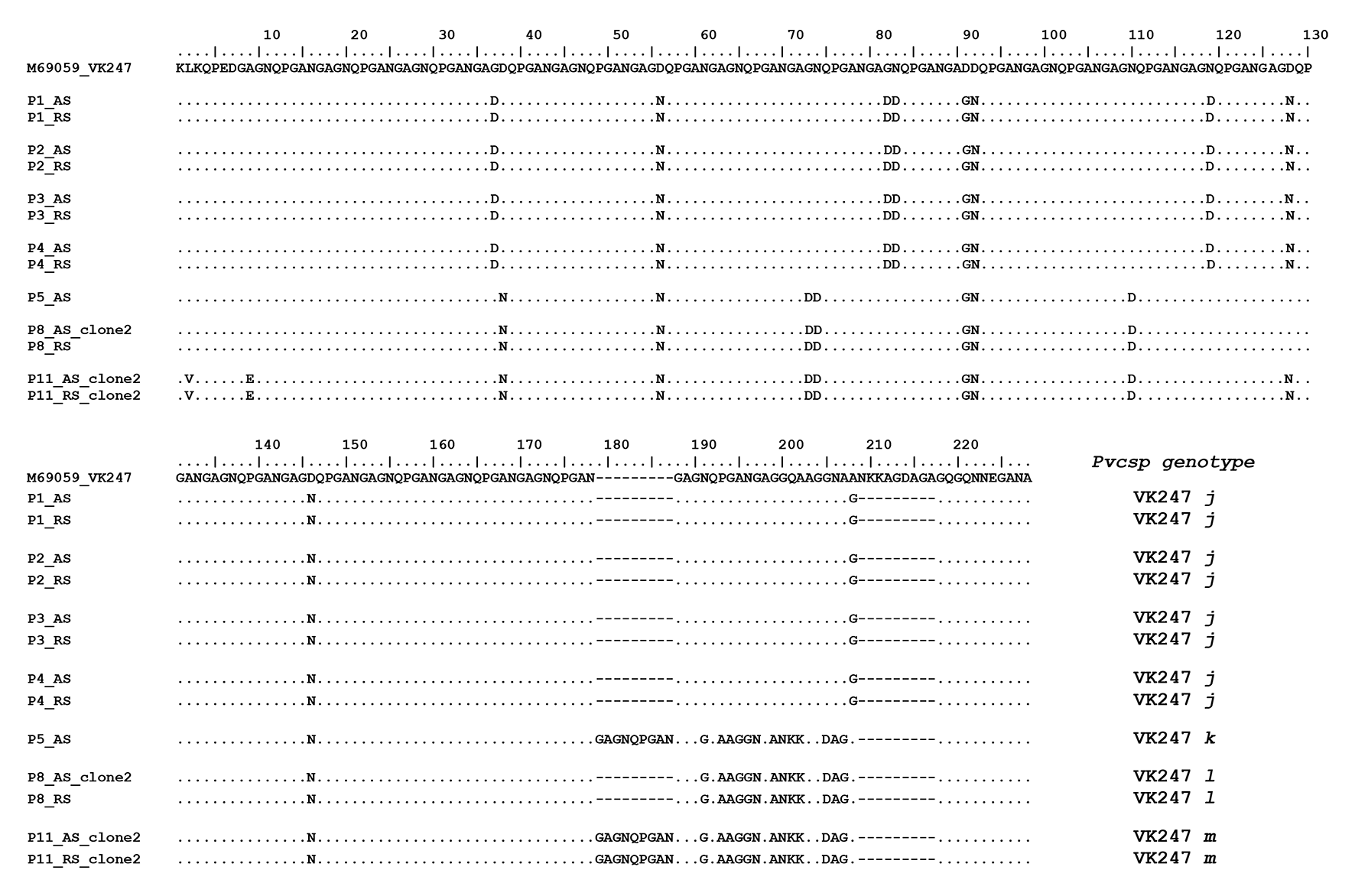


**Figure S3** Alignment of the predicted amino acid sequence of *Pvcsp* fragments bearing the VK247 repeat type in primary and relapse isolates. The alignment is based on the *Pvcsp* sequence of PNG strain (accession number M69059). Dots and dashes indicate identical residues and deletion, respectively. P, patient; AS, admission sample; RS, relapse sample.
